# Naringenin Decreases Retroperitoneal Adiposity and Improves Metabolic Parameters in a Rat Model of Western Diet-Induced Obesity

**DOI:** 10.3390/metabo15020109

**Published:** 2025-02-08

**Authors:** Gabriela López-Almada, J. Abraham Domínguez-Avila, Rosario Maribel Robles-Sánchez, Jonathan Arauz-Cabrera, Gustavo Martínez-Coronilla, Gustavo A. González-Aguilar, Norma Julieta Salazar-López

**Affiliations:** 1 Facultad de Medicina de Mexicali, Universidad Autónoma de Baja California, Dr. Humberto Torres Sanginés, Centro Cívico, Mexicali 21000, BCN, Mexico; gabriela.lopez.almada@uabc.edu.mx (G.L.-A.);; 2 SECIHTI—Centro de Investigación en Alimentación y Desarrollo A.C., Carretera Gustavo Enrique Astiazarán Rosas No. 46, Col. La Victoria, Hermosillo 83304, SO, Mexico; abrahamdominguez9@gmail.com; 3 Departamento de Investigación y Posgrado en Alimentos, Universidad de Sonora, Blvd. Luis Encinas y Rosales, Col. Centro, Hermosillo 83000, SO, Mexico; 4 Centro de Investigación en Alimentación y Desarrollo A.C., Carretera Gustavo Enrique Astiazarán Rosas No. 46, Col. La Victoria, Hermosillo 83304, SO, Mexico

**Keywords:** phenolic compounds, hunger, satiety, cholecystokinin, ghrelin, obesity, high-fat diet

## Abstract

**Background:** Obesity is a multifactorial disease with detrimental effects on health and quality of life; unregulated satiety plays a crucial role in food intake and obesity development. Naringenin (NAR) has shown beneficial effects on lipid and carbohydrate metabolism, although its impact on adiposity and satiety remains unclear. This study reports a Western diet (WD)-induced obesity model in rats, wherein 100 mg/kg of NAR was administered as an anti-obesity agent for 8 weeks; oxidative stress, lipid profile, and satiety biomarkers were then studied, as well as in silico interaction between NAR and cholecystokinin (CCK) and ghrelin receptors. **Results:** NAR supplementation resulted in a significant decrease in retroperitoneal adipose tissue and liver weight, as compared to the untreated WD group (*p* < 0.05), potentially associated with a decreased feed efficiency. NAR also inhibited the development of dyslipidemia, particularly by reducing serum triglycerides (*p* < 0.05). NAR supplementation increased CCK serum levels in the basal diet group, an effect that was abolished by the WD (*p* < 0.05); likewise, no changes were determined on ghrelin (*p* > 0.05). In silico data shows that NAR is capable of interacting with the CCK and ghrelin receptors, which suggests a potential for it to modulate hunger/satiety signaling by interacting with them. **Conclusions:** We conclude that NAR has anti-obesogenic effects and may regulate CCK serum levels, although further research is still needed.

## 1. Introduction

Obesity is a major global health problem that results from complex interactions between multiple factors. One of the main lifestyle factors is poor dietary habits, which often leads to hyperphagia and, alongside low physical activity, results in a positive caloric imbalance that manifests in excessive accumulation and dysfunction of adipose tissue [1]. Adipose tissue secretes a variety of molecules that regulate and participate in multiple signaling pathways, thereby acting as an endocrine gland. When it suffers hypertrophy in the obese state, it becomes a source of pro-inflammatory cytokines and oxidative stress, which together contribute to the development of a chronic, low-grade dysfunctional state [2]. Because of this, obesity is accompanied by a chronic low-grade inflammatory state that is capable of initiating and promoting other chronic non-communicable diseases (NCDs); thus, the search for effective and preventive treatments for obesity is an ongoing research area.

Among the different alterations documented in the obese state, hyperphagia is thought to occur due to dysregulations in the hunger/satiety pathway [3].

Satiety regulation encompasses both central and peripheral mechanisms. Centrally, the arcuate nucleus (ARC) in the hypothalamus plays a key role in satiety regulation through two populations of neurons that exert opposite effects when stimulated: proopiomelanocortin (POMC)- and cocaine- and amphetamine-regulated transcript (CART)-expressing neurons, which have an anorexigenic effect (together with α-melanocyte stimulating hormone (α-MSH)); and Agouti-related peptide (AgRP)/neuropeptide Y(NPY)-expressing neurons, both of which are potent orexigenic neuropeptides. Other structures, such as the nucleus tractus solitarius (NTS), brainstem, and vagal afferents also play a role. Both neuron populations receive centrally and peripherally derived signals to regulate feeding, creating a complex interplay. Peripheral signaling from the gastrointestinal tract (GIT) and adipose tissue, participate in this regulation through hormones and peptides that function as endocrine signals.

In the short term, AgRP neurons receive feeding-related signals derived from available nutrients in the GIT, which stimulate the release of enterohormones that relay this information. Subsequently, energy density and the activation of mechanoreceptors function as signals that promptly inhibit AgRP neurons. Leptin plays a main long-term regulatory role, as it is synthesized and secreted in proportion to adipose tissue mass and inhibits these neurons. The signals are collectively responsible for maintaining energy homeostasis [4].

Diet-induced obesity (DIO) induces leptin resistance on AgRP neurons, evidenced by failure to induce pSTAT3 in obese animals or downregulation of the activity of said neurons [3]. The activity of AgRP neurons in vivo during the development of DIO showed that it attenuates neural responses in a nutrient-specific manner, with blunted AgRP neuron responses to fat, but not glucose or protein [4].

Various GIT-derived hunger/satiety signals are known, such as cholecystokinin (CCK) [5] as one of the main satiety-inducing (anorexigenic) hormones, and ghrelin [6] as the main hunger-inducing (orexigenic) hormone. These and other compounds are released in response to nutritional stimuli (among others) and together act as the main short-term regulatory mechanism (minutes to hours) of hunger/satiety through vagus nerve afferents that transmit to the NTS in the brainstem. GIT-derived hormones are accompanied by long-term regulation from adipose-derived peptides, thereby allowing for precise energy balance regulation. All of these signals are integrated into the central nervous system (CNS), specifically, in the ARC of the hypothalamus, where neuron populations herein produce neuroactive compounds that result in a net orexigenic or anorexigenic effect, as previously stated. In the obese state, any part of the signaling pathway may become disrupted, which hinders returning to an adequate energy balance and may further promote its increase.

CCK is released by I cells of the duodenum and proximal jejunum, mainly in response to dietary fat and protein, and aids in their digestion by stimulating the release of bile from the gallbladder and pancreatic enzymes. In addition to its role in digestion, CCK also acts on vagal afferent neurons which, through the NTS, exert a central anorexigenic effect. Vagal endings in the stomach also respond to CCK, which relaxes gastric smooth muscle and inhibits gastric emptying [7]. Other molecules also respond to CCK, for example, the serum concentration of the anorexigenic hormone glucagon-like peptide-1 (GLP-1) increases, while that of ghrelin decreases, thus, the net anorexigenic effects of CCK are further amplified through its effects on said molecules. CCK exerts these effects by binding to the CCK receptors CCK1(CCK-A) or CCK2(CCK-B), which are G-protein coupled receptors. Several studies have concluded that the CCK-A (CCK-1) receptor is involved in regulating satiety, since its activation initiates satiety signaling to the hypothalamus via the vagus nerve [8]. The postprandial CCK increase tends to be rapid and high in lean individuals, reaching its maximum plasma concentration 15 min after feeding, thereby acting as a short-term satiety signal. In contrast, postprandial CCK concentration tends to remain elevated for longer or is decreased in obese individuals [9], which appears to be evidence of CCK resistance due to a decrease in the response of its receptor [10].

Ghrelin is synthesized and secreted primarily by gastric X/A cells and is known as the main orexigenic peptide. In healthy individuals, the circulating concentration of acylated ghrelin (its main active form) increases gradually during fasting and becomes maximum before feeding begins. Its concentration decreases in response to nutrient ingestion and, therefore, hunger also decreases with it in order to regulate short-term food intake. The effects of ghrelin on long-term adiposity have also been documented [11], and are apparently exerted by activating the sympathetic nervous system to increase fat deposition in adipose tissue and liver [12]. After being released into the peripheral circulation, ghrelin exerts its effects through vagus nerve signaling, in addition to directly in the CNS by crossing the blood–brain barrier and by in situ expression therein, where it binds to its receptor (growth hormone secretagogue receptor, GHSR). The GHSR, a 7 transmembrane G protein-coupled receptor, is expressed in the ARC’s AgRP/NPY-expressing neurons that, upon activation, increase the firing rate that contributes to promoting hunger in the individual [13,14]. It has been shown that the effects of ghrelin become dysregulated in the obese state, either through alterations in its concentration or a failure to decrease with food consumption [15]. Interestingly, this dysregulation has been mainly associated with decreased ghrelin mRNA in the hypothalamus, rather than changes in its gastric production [16,17]; in this way, the activation of AgRP/NPY neurons in the ARC is suppressed. These alterations are the result of a suppression of the ghrelin axis or “ghrelin resistance”, which is usually reversible with weight loss [18].

DIO causes central and peripheral resistance to ghrelin, which is associated with the inflammatory process present in obesity. Resistance to central administration of ghrelin results from a suppressed expression of the ghrelin receptor in AgRP/NPY neurons [18]. This also disrupts ghrelin signaling via the nodose ganglion and vagal afferents [19]. Furthermore, DIO has been associated with the desensitization of ghrelin-producing cells, resulting in lower circulating levels of ghrelin, downregulation of its mRNA expression, and reduced blood–brain barrier ghrelin transport [19]. This resistance is reversible with weight loss and a calorie-restricted diet.

Additionally, the macronutrient composition of a WD directly impacts ghrelin secretion, as dietary triglycerides, fatty acids, and hyperglycemia lower ghrelin levels [20]. This suggests a potential mechanism for detecting glucose availability by ghrelin-producing cells, as well as lipid receptors for short and long-chain fatty acids [21].

**Figure 1:**
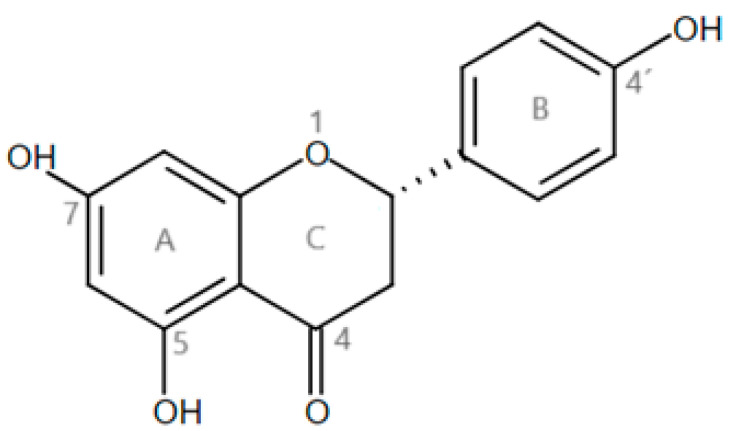
Chemical structure of naringenin (NAR, molecular weight 272.25 g/mol), the aglycone of the flavonoid naringin, which was studied in the present work.

Some bioactive compounds from foods have been shown to have an anti-obesogenic effect by modulating hormones involved in the hunger/satiety pathway, for example, by up-regulating mRNA expression and plasma GLP-1 concentration [22], stimulating the release of CCK, GLP-1, and peptide tyrosine–tyrosine (PYY) [23], as well as stimulating CCK release in vitro [24]. However, it should be mentioned that the mechanisms involved are not yet fully elucidated. Naringenin (NAR) is the aglycone form of the flavonoid naringin (Figure 1), whose consumption has shown multiple benefits on lipid and carbohydrate metabolism. In this regard, Assini et al. [25] used a high-fat diet model supplemented with NAR, and demonstrated that it prevented weight gain and improved serum triglycerides, glucose tolerance, and hyperglycemia [25]. Weight gain prevention by NAR has also been demonstrated in vivo [26,27]. In a previous review, we identified that NAR has modulation capabilities of different obesity-inducing factors, including peripheral and molecular markers involved in adipogenesis, thermogenesis, dyslipidemia, oxidative stress, and inflammation; however, the understanding regarding its effects on the hunger/satiety pathway requires further studies [26,27]. While some studies report reductions in food intake [28], others show only a tendency toward a decrease in food intake [26]. The differences in genetic background across these models may influence the results; however, this effect still needs to be described in a DIO model. On the other hand, it appears to participate in the satiety pathway, according to its ability to increase CCK concentration, as reported in vitro [29], and appears to interact with and activate GHSR, as evidenced by an increase in intracellular calcium in vitro [30]. Based on the aforementioned evidence, the impact of NAR consumption on the hunger/satiety pathway seems to be promising, although multiple actions remain unexplored, such as the mechanisms by which it interacts with the CCK (CCKR) and ghrelin receptors (GHSR). The potential role of NAR in regulating serum CCK and ghrelin, further influencing anorexigenic responses, requires particular investigation.

The present research focused on evaluating the in vivo effects of NAR on plasma CCK and ghrelin levels, two important peptide hormones involved in the hunger/satiety pathway, as well as its effects on metabolic parameters associated with obesity in a rat model of diet-induced obesity. We then used molecular docking analyses to analyze the interaction of NAR with the human CCKR and GHSR, in order to study interactions of the different ligand conformations with the active site of the receptors through predictive interactions, which may help to identify the interaction mechanism of this compound and, therefore, clarify its potential effect to act on the hunger/satiety pathway. Our data are intended to contribute to the understanding of the role of NAR on the hunger/satiety pathway.

## 2. Results

### 2.1. NAR Mitigates an Increase in Retroperitoneal Adipose Tissue

**Figure 2:**
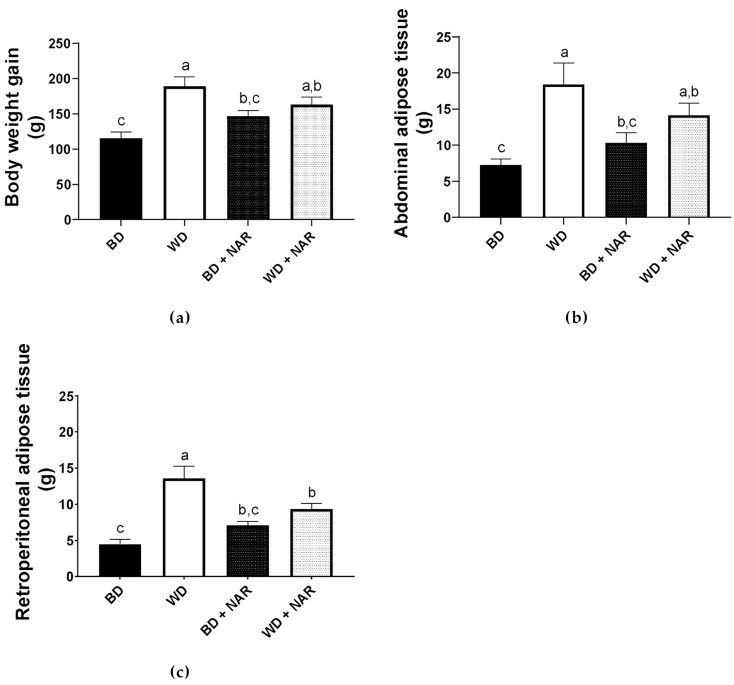
Effect of naringenin (NAR) on total body (**a**), abdominal (**b**), and retroperitoneal (**c**) adipose tissue weight after 8 weeks of treatment. BD: basal diet; WD: Western diet; WD + NAR: Western diet + naringenin; BD + NAR: basal diet + naringenin. Data are presented as mean ± standard error of the mean (SEM) (n = 6 per group). Different letters indicate significant differences between groups (*p* < 0.05). NAR: 100 mg/kg body weight.

Body weight was periodically measured, while adipose tissue was excised and weighed at the end of the experimental period. Figure 2a shows that the WD group had a higher body weight gain (189.00 ± 13.66, g), as compared to the BD group (115.58 ± 8.88, g; *p* < 0.05) which results in a 38.8% gain difference. NAR supplementation failed to significantly prevent WD-induced weight gain in this time period.

Figure 2b,c show adipose tissue weight. It is apparent that the abdominal (visceral) and retroperitoneal adipose tissue weights of the WD group were 60.6% and 67.0% higher, respectively, as compared to the BD group. NAR supplementation (WD + NAR) prevented retroperitoneal adipose tissue weight gain by 31.2%, although it failed to significantly prevent abdominal adipose tissue weight gain.

According to these results, the body weight gain observed in the WD group appears to be primarily attributed to fat deposition in the abdominal region in response to the diet. NAR treatment showed anti-adipogenic properties, but these were localized to the secondary deposit (retroperitoneal) instead of the main one (abdominal/visceral), thereby revealing different effects between the distinct adipose tissues, which could be due to physiological differences between each one.

### 2.2. NAR Exerts Hepatoprotective Effects, According to Gross Liver Features and Histology

**Figure 3:**
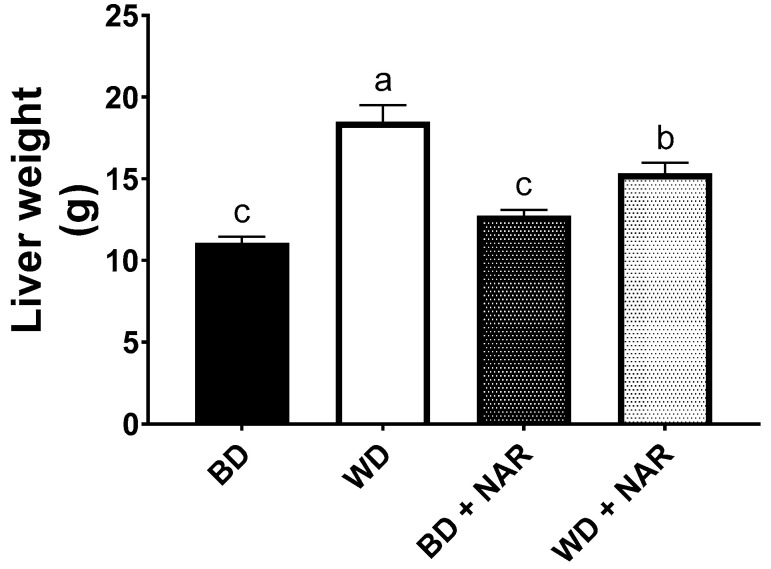
Effect of naringenin (NAR) on liver weight after 8 weeks of treatment. BD: basal diet; WD: Western diet; WD + NAR: Western diet + naringenin; BD + NAR: basal diet + naringenin. Data are presented as mean ± standard error of the mean (SEM) (n = 6 per group). Different letters represent significant differences between groups (*p* < 0.05). NAR: 100 mg/kg body weight.

Figure 3 shows liver weights after the experimental period. It is apparent that the livers of the WD group had a significantly higher weight (18.50 ± 1.01 g), as compared to the BD group (11.08 ± 0.39 g) (*p* < 0.05). NAR supplementation effectively hindered liver weight gain by 17%, despite the obesogenic diet (WD + NAR: 15.33 ± 0.65; *p* < 0.05 vs. WD).

**Figure 4:**
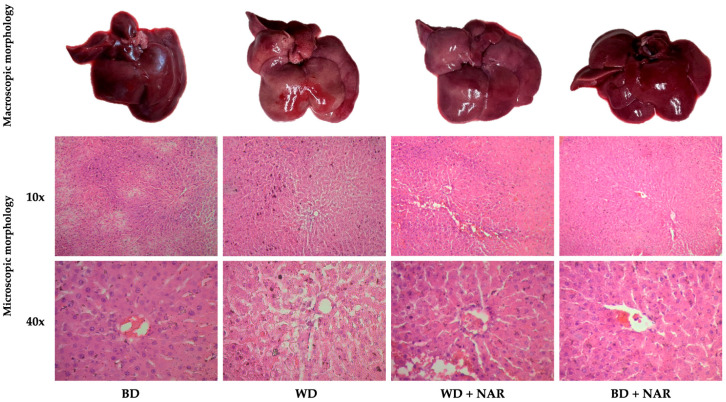
Macroscopic morphology of whole liver and microscopic morphology (H&E staining) in liver tissue at the end of 8 weeks of 100 mg/kg body weight NAR administration. H&E: hematoxylin and eosin staining. 10× and 40× magnification by light microscopy.

Representative livers (macroscopic morphology) and micrographies (microscopic morphology) from each experimental group are shown in Figure 4. Regarding macroscopic evaluation of the liver, the BD group had a normal appearance characterized by a bright red color. The WD group exhibited a slightly bigger liver and a subtle but noticeable pale color, suggestive of lipid accumulation. The external characteristics of the WD+NAR group livers were mostly healthy and similar to those of the BD group. These findings appear to be secondary to a reduced lipid deposition, thereby strengthening the anti-adipogenic properties of NAR. To confirm this, further microscopic evaluations were conducted.

Histological analyses using hematoxylin and eosin (H&E) staining allow to identify differences between groups, such as lipid accumulation (steatosis), which indicates ectopic and pathological lipid storage in response to excessive dietary energy intake [31]. H&E staining and light microscopy of liver tissue from the BD group revealed a regular cellular morphology with homogeneous cytoplasm and delimited sinusoids. In contrast, the WD group exhibited edematous and disorganized hepatocytes, characterized by increased intercellular spaces and accumulation of lipid droplets/vacuoles (steatosis), both in macro- and microvesicular forms. Treatment with NAR attenuated WD-induced hepatosteatosis, as evidenced by the WD + NAR group, which had a more regular and uniform morphology without overt lipid vacuoles. This suggests that NAR significantly hinders hepatic fat accumulation caused by a WD. The BD + NAR group exhibited a structure similar to that of the BD group, with no pathological changes, potentially confirming the absence of hepatotoxicity.

### 2.3. NAR Mitigates Dyslipidemia

**Figure 5:**
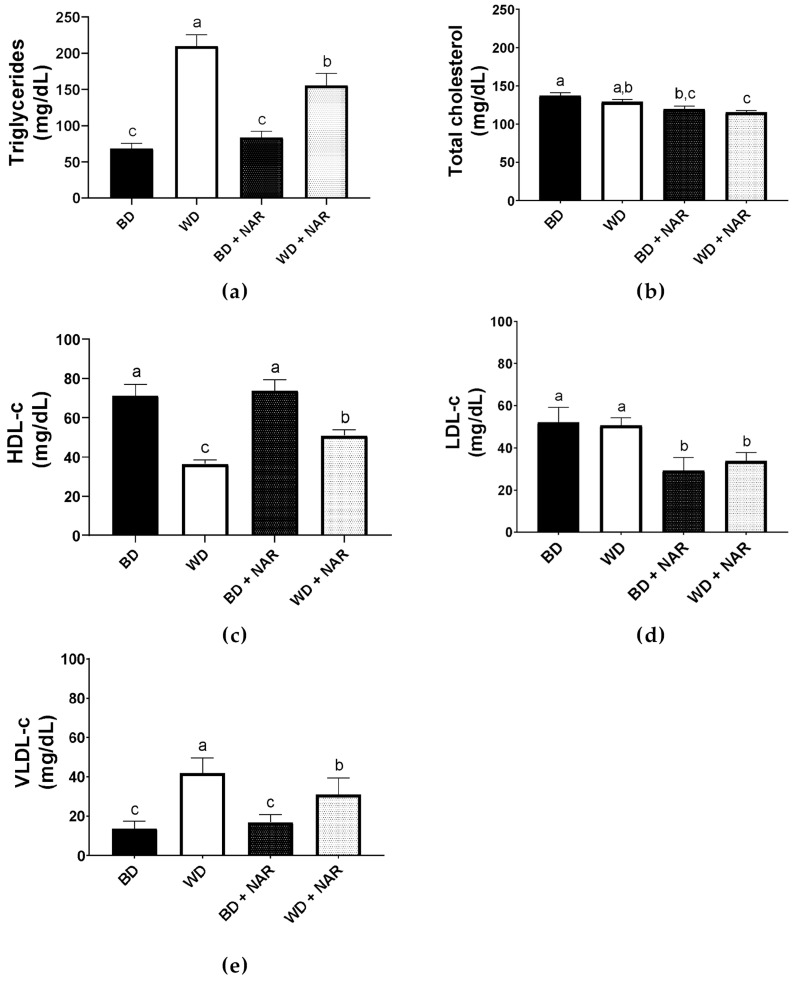
Effect of naringenin (NAR) on total serum triglycerides (**a**), cholesterol (**b**), HDL-c (**c**)**,** LDL-c (**d**), and VLDL-c (**e**), and levels after 8 weeks of treatment. BD: basal diet; WD: Western diet; WD + NAR: Western diet + naringenin; BD + NAR: basal diet + naringenin. Data are presented as mean ± standard error of the mean (SEM) (n = 6 per group). Different letters represent significant differences between groups (*p* < 0.05). NAR: 100 mg/kg body weight.

The serum lipid profile is shown in Figure 5, where triglycerides (TG), total cholesterol, HDL-c, LDL-c, and VLDL-c levels were measured. The WD group developed hypertriglyceridemia, as compared to the BD group (*p* < 0.05), an effect that was expected due to the well-known role of WD diets on inducing and promoting dyslipidemia. NAR supplementation partially and significantly prevented the development of hypertriglyceridemia in this model, according to a 26% reduction in TG (Figure 5a).

No changes were documented in total cholesterol between the BD and WD groups, but a significant difference was observed in both experimental diets when supplemented with NAR (*p* < 0.05) (Figure 5b). Regarding HDL-c, the WD group showed a decrease, as compared to the BD group (*p* < 0.05). The WD + NAR group had a 28% increase in HDL-c levels (50.9 ± 3.10; *p* < 0.05 vs. WD) (Figure 5c).

There was no significant difference between LDL-c of the BD and WD groups; however, NAR supplementation exerted protective effects on both experimental diets, by significantly decreasing LDL-c levels by 33% in the WD + NAR group (vs. WD, *p* < 0.05), and by 43.9% in the BD + NAR group (vs. BD, *p* < 0.05) (Figure 5d).

The WD group had increased VLDL-c, as compared to the BD group (*p* < 0.05). NAR supplementation in the WD group partially prevented the increase in this lipoprotein by 25% (*p* < 0.05 vs. WD) (Figure 5e).

The changes in the lipid profile induced by the WD align with blood lipid alterations that are commonly observed in the obese state, such as an increase in atherogenic biomarkers (e.g., TG and LDL-c) and a decrease in antiatherogenic lipoproteins (e.g., HDL-c). Pearson analyses showed a positive correlation between body weight gain and liver weights (r = 0.87, *p* = <0.0001), retroperitoneal adipose tissue (r = 0.86, *p* = 0.0001), and abdominal adipose tissue (r = 0.82, *p* = 0.0001), suggesting that body weight gain is due to the sum of liver and adipose tissue weights, secondary to increased dietary serum TG and their deposition in these organs.

NAR supplementation was apparently able to regulate these lipoproteins in spite of a WD, thereby providing an additional benefit to the previously discussed anti-adipogenic ones. It is therefore suggested that NAR favorably modulates the metabolism of both storage and circulating lipids.

### 2.4. Antioxidant Effect of NAR

**Figure 6:**
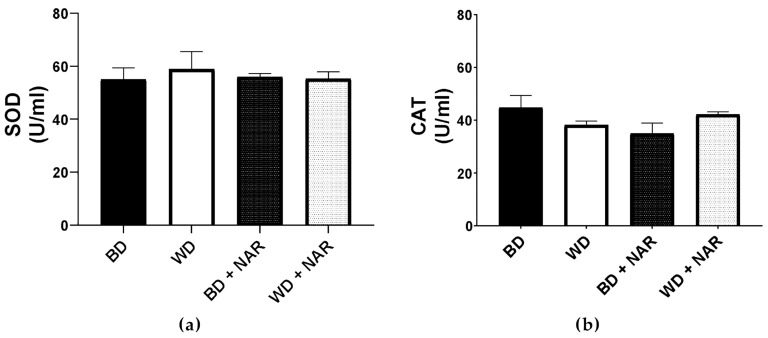
Effect of naringenin (NAR) on SOD (**a**) and CAT (**b**) activity after 8 weeks of treatment. BD: basal diet; WD: Western diet; WD + NAR: Western diet + naringenin; BD + NAR: basal diet + naringenin. Data are presented as mean ± standard error of the mean (SEM) (n = 6 per group). NAR: 100 mg/kg body weight.

Figure 6 shows the effect of NAR supplementation on the activities of serum superoxide dismutase (SOD) and catalase (CAT), which are two important enzymes of the antioxidant defense system and biomarkers of the oxidative state. It is apparent that no significant changes were detected in the activity of either enzyme.

### 2.5. Effect of NAR on Satiety

#### 2.5.1. Food Consumption

**Figure 7:**
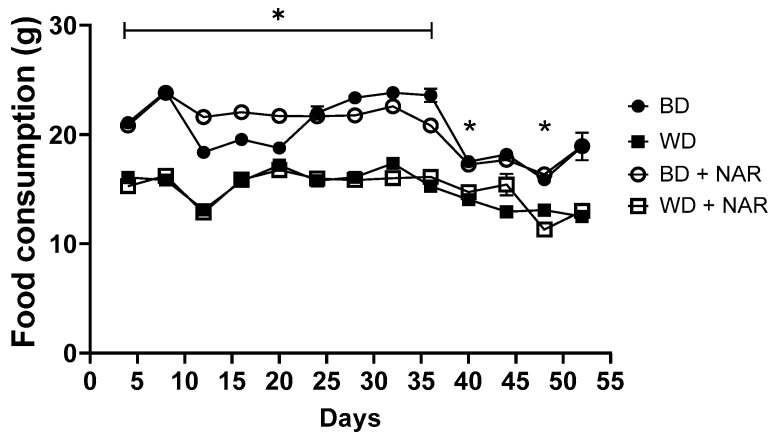
Effect of Naringenin (NAR) on food consumption during the 8-week treatment period. BD: basal diet; WD: Western diet; WD + NAR: Western diet + naringenin; BD + NAR: basal diet + naringenin. Data are presented as mean ± standard error of the mean (SEM) (n = 6 per group). * *p* < 0.05 between BD and WD groups with respect to time. NAR: 100 mg/kg body weight.

Studying the effects of NAR on dietary intake could unveil a novel mechanism by which it could regulate body weight and, consequently, exert an anti-obesogenic effect. Food consumption throughout the experimental period is shown in Figure 7. The food consumption pattern documented throughout the experimental period revealed a difference between BD and WD groups, which were expected results due to the differences in diet composition. NAR supplementation did not result in significant changes in food consumption throughout the experimental period (*p* > 0.05); it is therefore not possible to establish satiety through reduced food consumption due to the presence of NAR in the diet; further studies were conducted to clarify the effect of NAR on the hunger/satiety pathway.

#### 2.5.2. NAR Decreases Feed Efficiency

Feed efficiency was determined, which establishes the relationship between energy intake and adiposity, or the percentage of consumed feed stored as fat [32,33]. This makes it possible to determine an association between these two variables; results are shown in Table 1.

It is apparent that the WD group had a lower energy intake than the BD group, but it nevertheless had a higher efficiency than the BD group, which indicates the adipogenic effect of the WD. The addition of NAR did not result in any difference in energy intake; however, it significantly decreased feed efficiency in the WD + NAR group, while maintaining similar values when incorporated in the BD group (BD + NAR). This suggests that NAR is able to counter the adipogenic effect of a WD.

#### 2.5.3. NAR Modulates Peripheral Peptides Involved in the Satiety Pathway

**Figure 8:**
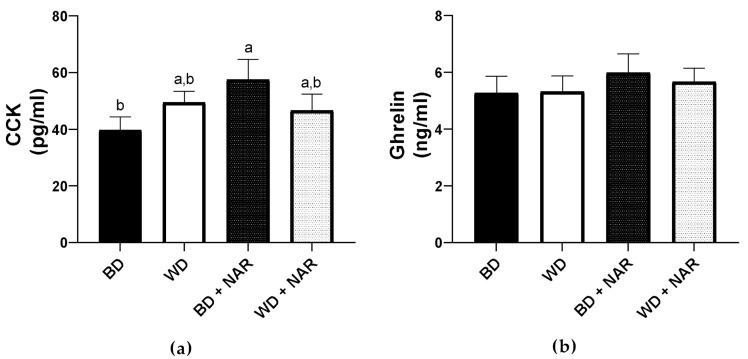
Effect of naringenin (NAR) on plasma cholecystokinin (CCK) (**a**) and ghrelin (**b**) and levels following 8 weeks of treatment. BD: basal diet; WD: Western diet; WD + NAR: Western diet + naringenin; BD + NAR: basal diet + naringenin. Data are presented as mean ± standard error of the mean (SEM) (n = 6 per group). Different letters represent significant differences between groups (*p* < 0.05). NAR: 100 mg/kg body weight.

The effects of NAR on peripheral peptides involved in the hunger/satiety pathway (CCK and ghrelin) were quantified; results are shown in Figure 8. NAR supplementation increased CCK concentration (*p* < 0.05) when administered alongside a BD (Figure 8a), but not WD. This could indicate that NAR’s regulatory effect on peripheral CCK levels is countered by the nutritional profile of the WD. Regarding ghrelin (Figure 8b), no significant differences were found between any groups, which is consistent with food consumption data. These results provide insight into NAR’s effect on enteroendocrine cells in vivo. To further elucidate NAR’s influence on the satiety pathway, a molecular docking analysis was performed to approach NAR’s effect on the CCK and ghrelin receptors.

#### 2.5.4. Molecular Docking with CCK and Ghrelin Receptors

A predictive molecular docking analysis of the different conformations of NAR with the CCK and ghrelin receptors was performed, in order to determine potential binding (according to conformational binding energy), and to determine the interaction potential between NAR and hormone receptors. M.O.E. (Molecular Operating Environment, 2022.02) software was chosen because it allows us to evaluate the diversity of interactions and conformations, as well as the binding affinity by means of the S-score and, therefore, compare possible interactions [34]. Results are shown in Table 2.

The results showed 3 possible conformations for NAR. The most negative S-score values with the best positions were selected; a negative S-score suggests the prediction of higher affinity values between the two molecules (ligand and receptor). Conformational analysis followed by molecular docking allowed us to observe that the characteristics of the flavonoid allow it to fold and position itself in the receptor.

**Figure 9:**
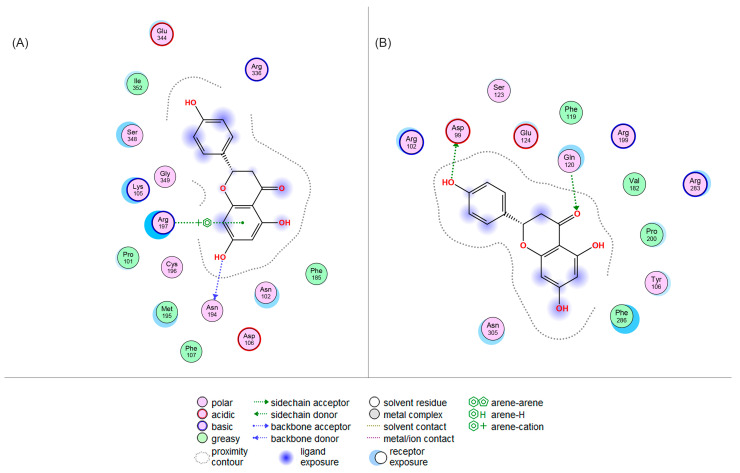
Two-dimensional conformation of the best interaction between (**A**) NAR and the cholecystokinin receptor (CCKR) as well as (**B**) NAR and the ghrelin receptor (GHSR). The proximity contour is shown by the dotted line. Hydrophilic residues are shown in purple, hydrophobic residues are shown in green, blue rings indicate basic groups, red rings indicate acidic groups, and black rings indicate neutral groups.

The interactions between NAR and CCKR are shown in Figure 9A. Docking revealed the best and main interactions between NAR and CCKR. The second-best position in the S-score was chosen with an S-score of −5.5540. Interactions revealed an arene–cation interaction between the Arg197 of the receptor and phenolic A ring of NAR, as well as a backbone donor interaction of the hydroxyl group on position 7 of the phenolic A ring with Asn194.

The interactions between NAR and GHSR are shown in Figure 9B. The third-best position was chosen with an S-score of −5.7476, with interactions between Gln120 and the carbonyl functional group at position 4 of the C phenolic ring, as well as the hydroxyl group on the 4’ position of the B phenolic ring with Asp99.

## 3. Discussion

The WD administered to the animals of the present study intends to simulate the high-fat, hypercaloric dietary pattern commonly consumed in the Western world. Such diets contribute to the development of chronic NCDs through the combination of high fat and sugar, and low fiber and antioxidants, as compared to a healthy diet [35,36]. We therefore induced obesity by administering a WD for 8 weeks, which resulted in an increased body weight, in addition to alterations of serum lipids, which are indicative of dyslipidemia that commonly occurs alongside obesity [37].

A recent study about administration of NAR in a co-amorphous solution demonstrated a significant prevention in fat-mass gain, improved lipid metabolism, and enhanced energy expenditure. However, no changes were observed in food intake and no additional studies were conducted to evaluate the role of NAR on satiety markers [38]. This highlights the need for further studies to evaluate the effect of NAR on the satiety pathway.

NAR supplementation has been shown to have a preventive effect on weight gain in vivo [39,40]. For example, Liu et al. [40] administered different NAR doses (25, 50, and 100 mg/kg) by oral gavage to Wistar rats, while simultaneously feeding them a high-fat diet. They report a reduction in body weight gain between the supplemented and unsupplemented groups at the 100 mg/kg dose after 4 weeks (*p* < 0.05). Although this experiment used the same dose as the present study, it was shorter than ours and had a lower fat percentage (27%), which could favor the decrease in body-weight gain reported by said authors. On the other hand, the study of Burke et al. [41] used a high-fat (42% of calories from fat) high-cholesterol diet, on obese LDLR^−/−^ mice and treated them with NAR at 3% *w/w* (weight percent). This resulted in a body weight loss of ~13% after the intervention, which was significant compared to the unsupplemented group. However, the supplementation period in this study was longer than the present one (12 weeks), which may have allowed for this change to become significant.

Total calorie intake on a WD is typically higher than that of a balanced diet, which results in greater weight gain due to the excess energy consumed that is stored as TG in adipose tissue. Even if the animals consume only their required caloric intake (no surplus of energy), the amount and type of fats and carbohydrates may still induce fat storage. For example, long-chain saturated fatty acids, like those found in lard, are not oxidized as efficiently as unsaturated fatty acids, making them easier to store as TG in adipose tissue [42]. Simple sugars like fructose are also highly adipogenic, and are able to impact not only adipose tissue but intrahepatic and circulating lipids as well [43]. In rats, an obesogenic diet has been associated with increased hyperplasia of subcutaneous adipose tissue, hypertrophy of visceral [44], and retroperitoneal adipose tissue [43]. Visceral adipose tissue is more vascularized, innervated, and metabolically active, while also having increased lipolysis and inflammatory potential [45]. It therefore participates more in the release of cytokines and fatty acids, which is why an increase in visceral fatty tissue has been more closely associated with increased mortality and cardiovascular risk than subcutaneous adipose tissue [46].

The ability of NAR observed in this study to prevent the accumulation of retroperitoneal adipose tissue is consistent with other studies. A summary of the principal findings reported herein is presented in Table 3. For example, Ke et al. [47] performed an intervention model in ovariectomized female C57BL/6J mice, using a standard diet supplemented with 3% NAR for 11 weeks. At the end of the study, a decrease in body weight was observed in the supplemented group, along with a decrease in total, intra-abdominal, and subcutaneous adiposity by 54, 59, and 50%, respectively [47]. Other studies have shown similar results regarding changes in adipose tissue [26,41]. Burke et al. [26] reported significantly decreased inguinal adiposity of up to 71% after dietary supplementation with NAR, as compared to the unsupplemented group. Similarly, another study reported that administration of 100 mg/kg NAR for 4 weeks reduced visceral and epididymal adipose tissue [40]. The results reported in these studies were in different types of adipose tissue (epididymal, inguinal, visceral, etc.), which may limit the direct comparability of the results, although there is consistency regarding the anti-adipogenic effects of NAR.

NAR has been reported to attenuate metabolic disturbances through AMPK activation; eight weeks of NAR administration in estrogen deficiency-induced obesity prevented body weight gain and suppressed visceral and subcutaneous white-adipose-tissue (WAT) accumulation. These findings were associated with increased levels of browning markers in WAT in vivo and in vitro, as well as AMPK phosphorylation which, through the modulation of mitochondrial biogenesis and thermogenesis, ameliorated adipocyte hypertrophy and promoted adipocyte browning. This effect was blocked by an AMPK inhibitor and AMPK knockdown [48].

NAR appears to induce browning and fatty acid oxidation in human white adipocytes (in vitro) by upregulating mRNA expression of proteins involved in these processes, including UCP1, PPARγ coactivator PGC1α, and carnitine palmitoyltransferase 1B. This regulation seems to be dependent on the activation of AMPK, a metabolic regulatory protein that increases catabolic processes when activated, as evidenced by an increase in AMP phosphorylation following NAR treatment [49]. Additionally, the increased ATGL (adipose tissue lipase) mRNA in this same study suggests enhanced triglyceride hydrolysis and induction of lipolysis [49], further supporting the induction of catabolic processes. On the other hand, an in vivo study shows that reduced adiposity with NAR treatment was not associated with increased mRNA expression of browning markers or lipolysis-associated genes, such as UCP1, PPARα, or Pnpla2, but rather with an increase in energy expenditure [41], which suggests another unidentified mechanism by which NAR decreases adiposity.

Chronic consumption of a WD also triggers ectopic fat storage. Adipose tissue in the obese state is characterized by being hypertrophic and hyperplastic tissue with compromised functionality and potential ectopic storage, such as in the liver. This triggers an inflammatory process and the release of fatty acids, products of lipolysis, which allows an excess of fatty acids to reach the liver through the portal circulation, subsequently promoting the synthesis of intrahepatic triglycerides [50]. A number of key enzymes in the hepatic lipogenic pathway are also stimulated which, together with high dietary fructose, induces hepatic steatosis through de novo hepatic lipogenesis (DNL) [51]. All of these alterations contribute to an increased liver weight, as observed in this research.

The visible hepatic steatosis in the WD group could be associated with the characteristics of the diet, including fructose. The increase in serum fatty acids secondary to fructose consumption has been shown to be significant, even more than other sugary drinks like glucose and sucrose [52]. In this regard, Donnelly et al. [53] defined the sources of fatty acids and TG stored in the liver of obese patients with nonalcoholic fatty liver disease and showed that more than half (59%) of the TG and fatty acids come from adipose tissue lipolysis, with the remainder coming from de novo synthesis in the liver (26%) and from the diet (15%) [53]. These findings could explain the steatosis observed in the WD group, while NAR supplementation favored a lower accumulation of TG, potentially secondary to decreased DNL. Hepatosteatosis is histologically characterized by the accumulation of fat in the form of intrahepatic TG-containing droplets or vesicles. According to their size, vesicles can be referred to as microvesicular or macrovesicular. Microvesicular steatosis can be initially observed as ballooning or edematous hepatocytes, characterized by a reticulated or foamy cytoplasm due to small fat droplets. Steatosis lesions usually predominate in zone 3 of the hepatic acinus, characterized by the presence of the central vein [54]. The benefits of NAR in preventing hepatic steatosis have been reported previously; for example, Ke et al. [47] demonstrated a significant decrease in total lipid and TG content in the liver of an in vivo model supplemented with NAR, as compared to the unsupplemented group. Similarly, a decrease in hepatic TG of 58–82% was reported at the end of a 12-week intervention period in a high-fat diet model [41], which is consistent with our data. This suggests a potential anti-adipogenic and hepatoprotective effect exerted by NAR supplementation, and its mechanisms appear to be related to regulating gene expression of lipid metabolic pathways, as multiple in vivo studies have reported that NAR treatment led to a decrease in liver mRNA expression of ACC, FAS, HMGCR, Srebp1c, and Scd1. In contrast, NAR treatment increased cpt1a, PPARα, Pgc1α, and Srebf1 [26,47,55,56], which seems to occur in a dose-dependent manner [55,56]. Additionally, there was an increase in hepatic ATGL/PNPLA, a rate-limiting lipase involved in the hydrolysis of triglycerides [26]. Overall, this suggests that NAR reduces hepatic DNL by downregulating gene expression of lipogenic enzymes, while also upregulating those involved in hepatic fatty acid oxidation, mitochondrial biogenesis, and enhanced lipolysis. This was further confirmed by an increase in plasma beta-hydroxybutyrate, a marker of fatty acid oxidation [26].

NAR has also been shown to promote AMPK mRNA expression and AMPK phosphorylation in vitro, which suggests that NAR’s ability to modulate lipid metabolism is associated with the AMPK pathway [55]. Another study reports that NAR exerts its anti-lipogenic effects by downregulating the NLRP3/NFκB signaling pathway in vitro, in a NLRP3-dependent manner [57], which suggests that the lipid accumulation process is closely associated with the inflammatory response.

The WD induces changes to the lipid profile, including increased TG and VLDL-c, and reduced HDL-c levels. The increase in TG in the WD group may be attributed to fructose, which has been linked to hypertriglyceridemia in studies using similar concentrations (23%) over a 2-week period [52]. Another study used 16% fructose and found a significant increase in circulating TG after just 24 h of consuming it, highlighting its short-term lipogenic potential [58]. The protective effects of NAR observed on these parameters are consistent with findings from other studies, such as a decrease in TG levels of up to 46% reported in a genetically modified animal model (LDLR^−/−^), which was fed a diet supplemented with 3% NAR for 8 weeks [26]. Similarly, a reduction of more than 50% in TG was observed in a high-fat diet-induced obesity model [41]. The mechanism by which NAR manages anti-dyslipidemic effects suggests that it may be secondary to the increase in hepatic fatty acid oxidation or the reduction in hepatic DNL, as stated above. NAR has also demonstrated the ability to reduce and prevent dyslipidemia by increasing fatty acid oxidation by activating PPAR-γ and preventing SREBP1-mediated lipogenesis by reducing hyperinsulinemia and decreasing VLDL-c [56].

It has been previously reported that NAR exerts antioxidant effects by neutralizing free radicals, a property linked to its chemical structure that contains hydroxyl groups responsible for hydrogen ion transfer and free radical scavenging [59]. Beyond this direct antioxidant activity, NAR also modulates the endogenous antioxidant system, including the nuclear transcription factor erythroid 2 (Nrf2) [60,61,62,63]. Key endogenous antioxidants like SOD and CAT play crucial roles in protecting cells from oxidative stress. SOD facilitates the dismutation of superoxide radicals into molecular oxygen and hydrogen peroxide, while CAT catalyzes the conversion of hydrogen peroxide into water and molecular oxygen. The activity of both enzymes is often upregulated in response to oxidative stress, which results from an excess of reactive oxygen species (ROS), often seen in obesity. However, our results showed no differences in the activity of plasma SOD or CAT in response to NAR supplementation, which may be attributed to several factors, including an insufficient NAR dose to elicit a systemic antioxidant response or a potentially short experimental period. However, the WD also exerted no significant changes in their activity.

Nguyen-Ngo et al. [64] used an in vivo model and studied the expression of these antioxidant enzymes in gestational diabetic mice treated with NAR (50 mg/kg body weight, administered intraperitoneally for 18 days). In visceral adipose tissue, a significant reduction in CAT gene expression was observed (*p* < 0.05), while there was an increase in SOD gene expression in subcutaneous adipose tissue (*p* < 0.05) [64]. Although these results suggest NAR’s potential to modulate these enzymes at the gene level, serum or systemic enzyme levels were not evaluated, and the animals were not subjected to different diets. In contrast, Liu et al. [40] evaluated serum SOD levels in Wistar rats fed a high-fat diet (27% fat, 41% carbohydrates) for 16 weeks. The high-fat diet group showed lower serum SOD levels, as compared to the control group (*p* < 0.05). Treatment with NAR at 50 and 100 mg/kg doses for the last 4 weeks of the study significantly increased serum SOD levels (*p* < 0.05 vs. high-fat diet). Compared to our study, the main difference with the work of Liu et al. [40] was a longer experimental period. Our findings are consistent with the study of Kobi et al. [65] who used a dietary intervention model with Wistar rats fed a high-fat, high-sucrose diet (HFHS; lipids 37.4%, carbohydrates 43.4%) for 20 weeks, and also reported no significant differences in serum SOD and CAT activities, suggesting that the dietary intervention did not induce oxidative stress in this model [65]. The macronutrient composition of the HFHS diet in their study is similar to the one used in the present work. Kobi et al. [65] suggest that the lack of hyperglycemia and hyperleptinemia in their model could explain the absence of changes in these oxidative stress markers, as well as the absence of other comorbidities. In contrast, our model did exhibit comorbidities, such as dyslipidemia, over an 8-week period, which may be attributable to the addition of fructose in the diet, potentially exerting an additive effect on the development of metabolic derangements. These differences warrant further investigation into the oxidative impact of the diet and the potential antioxidant properties of NAR at different doses, particularly at the tissue level (e.g., in adipose tissue) through tissue-specific OS markers, including MDA, in both long- and short-term models like ours.

Regarding food intake, one of the main determinants is the onset of satiety, which is triggered by signals generated during feeding and leads to the termination of eating. NAR supplementation did not alter food intake in either of the two diets; however, daily food consumption data indicated that the WD groups consumed less food and, therefore, a lower energy intake, as compared to the BD groups. This could be attributed to the characteristics of the WD, which is more energetically dense per gram given due to its high fat and carbohydrate content. Although the WD group consumed less food and fewer kilocalories, it had a higher feed efficiency which, combined with its sedentary environment, resulted in the accumulation of excess energy in the form of fat, culminating in a significant increase in body weight compared to rats exposed to a BD. This increase in weight was observed exponentially over time and was secondary to an increase in adipose tissue and liver tissue. Since NAR did not significantly alter dietary consumption, changes in adiposity and the lipid profile are likely to have occurred in response to changes in concentration and/or decreased resistance to hormones (e.g., leptin), energy expenditure and/or storage (e.g., uncoupling proteins), gut microbiota, or other variables that shifted the animals’ feed efficiency from energy storage to energy expenditure. This phenomenon has been reported in various models; for example, Yao et al. [66] report that it is possible to perform isocaloric dietary replacements and still obtain significant weight loss in human subjects. Additional studies are required in order to determine the precise mechanisms by which the changes documented in the present study occurred.

Rinde et al. [25] suggest that one mechanism underlying the decrease in adipose tissue storage is a decrease in feed efficiency. Our findings are consistent with this, as the addition of NAR significantly reduced feed efficiency values, as compared to the unsupplemented WD group, which coincided with a decrease in adipose tissue weight in the WD + NAR group. This suggests that the decrease in feed efficiency observed in the WD + NAR group, despite similar caloric intake and food consumption as that in the WD group, is due to a decrease in adipose tissue storage in response to NAR supplementation. This could indicate a protective or anti-obesogenic effect of NAR. The mechanism by which fat storage is reduced remains unclear; it may be secondary to increased energy expenditure [67] or by preventing TG synthesis and deposition. Further studies are needed to clarify this point.

The WD and BD diets used in our study shared a similar micronutrient composition, such as vitamins and minerals, which minimizes bias in the interpretations of results. This enables an objective evaluation of obesity development driven by the macronutrient (fats and carbohydrates) composition of the diet [44]. This could lead to a lower food intake in the WD groups as a compensatory mechanism to counterbalance the increased energy intake perceived by the central and peripheral regulatory systems of the hunger/satiety axis, thereby helping to maintain energy homeostasis and prevent excessive weight gain. It is noteworthy that a limitation in this study arises from the lack of a quantitative record of fructose intake (being ad libitum throughout the study), which limits a precise estimation of the daily energy intake of rats in the WD and WD + NAR groups.

Previous studies have identified structural characteristics of flavonoids that confer them the ability to interact with certain receptors, such as the presence of hydroxyl groups [68]. NAR is an amphipathic molecule due to its three hydroxyl groups that confer it a polar and hydrophilic behavior, while the aromatic rings are considered non-polar and hydrophobic [69]. This makes it possible for NAR to interact with polar, non-polar, charged, or uncharged amino acids of multiple proteins, including hormone receptors, potentially allowing it to directly modulate multiple physiological processes. The action of CCK and its receptor contributes to stimulating insulin secretion and delaying gastric emptying [7], in addition to regulating satiety, and seem to also depend in part on the co-secretion of other peptides like GLP-1 and leptin, since they have been shown to exert a synergistic or additive effect by promoting satiety [5]. Together, these hormones and their collective effects induce an anorexigenic effect.

Our in silico analysis selected the second-best molecular docking position between NAR and CCKR, as determined by the S-score and the interactions between NAR and the receptor, guided by the interactions between sulfated CCK-8 (its highest affinity natural ligand) with CCKR. In this regard, Liu et al. [70] evaluated the cryo-electron microscopy structures of the CCK-A receptor and sulfated CCK-8. The receptor used by the authors includes the extracellular and transmembrane domains, along with its coupling to G proteins (PDB ID: 7EZH, 7EZK, 7EZM). In contrast, our study utilized a receptor that consists of the extracellular and transmembrane domains (PDB ID: 7MBX). The study of Liu established that Arg197 is a determinant for discriminating between sulfated and non-sulfated CCK, making it crucial for its recognition and binding [70,71]. The study also described interactions in the binding pocket of the CCK-A receptor, which is involved in the recognition of sulfated CCK-8, particularly the extracellular portion through Asp333, His210, Tyr360, Arg197, and Ser348. Critical residues for CCK-AR activation like Arg336, Asp333, Ala343, Leu347, and Ser348 were also identified [70]. These residues and position reported by Liu et al. [70] coincide with ours, in which NAR was in proximity with other residues other than Arg197, such as Arg336 and Ser348, that are described as essential and critical for the recognition and activation of the endogenous ligand CCK-8. The evidence therefore suggests that NAR is capable of interacting with CCKR, in a manner similar to CCK-8, making it possible for it to act as an agonist, thereby eliciting direct anorexigenic effects through this mechanism. No in silico or molecular docking studies of NAR with the CCKR have been identified to our knowledge, which suggests the need for further analyses.

Ghrelin exerts its effects partly by signaling via the vagus nerve and directly in the CNS by crossing the blood–brain barrier, where it binds to GHSR. This receptor is expressed in AgRP/NPY neurons of the ARC, and exerts its orexigenic effects upon binding of peptide ligands and subsequent activation [13,14]. The ability of NAR to activate GHSR has been previously reported in vitro [30] (PubChem CID: 134817268). The third-best position between NAR and GHSR was selected based on the interactions between GHSR and GHSR agonist molecule HM01 (PubChem CID: 134817268), in which the fifth-best position showed an interaction with Asp99 (S-score −7.0356). This demonstrates that the NAR used in this study has an approximate affinity (−5.7476) for the receptor, similar to that of the original agonist (−7.0356). Asp is an acidic amino acid, which behaves as an electron acceptor of NAR. This is because Asp presents a negatively charged oxygen in its structure, which attracts the hydrogen of the hydroxyl group attached to the 4’ position of the B phenolic ring of NAR. On the other hand, Gln is a neutral polar amino acid, which behaved similarly to Asn194 in the NAR-CCK interaction through its -NH_2_ sidechain group, by donating hydrogen to the carbonyl group of the C phenolic ring of NAR, which is also polar. The outcome of this effect in vivo is unknown, although it is possible that it could be reflected in increased gastrointestinal motility [72,73] or as an orexigenic effect. Such information allows us to suggest that the acidity or basicity of the amino acid, the participation of electronegative elements, and the presence of hydroxyl groups in NAR and in the amino acids will favor the formation of possible interactions. To the best of our knowledge, the mechanism and effect of NAR on the CCK and ghrelin receptors have not been clarified and require further investigation.

Previous studies suggest that antagonism of GHSR signaling could ameliorate obesity by reducing food intake and promoting weight loss, as GHSR-null mice exhibited reduced fat deposition and increased energy expenditure, showing resistance to DIO [74]. Therefore, inhibiting GHSR signaling has been proposed as a therapeutic strategy for the treatment of obesity and related complications [75].

GHSR antagonists have been studied for their potential effects on weight reduction; however, the results have been contradictory, since some have been reported to induce weight gain [76]. A particularity of the GHSR is its high basal activity, which means it can become activated without the presence of its ligand ghrelin. This has led to the development of inverse ghrelin receptor agonists, which have been shown to significantly decrease receptor activity [77].

The GHSR, particularly GHSR1a, is found in the CNS (including the hypothalamus and brainstem), vagal afferents, adipose tissue, pancreatic cells, and other tissues. Its distribution and modulation play a role in determining its effects. Ghrelin receptor mRNA is expressed by ~94% of NPY neurons in the ARC, which highlights this region as a key site for short-term satiety regulation. GHSR1a is mediated by the Gαq/11, phospholipase C/inositol triphosphate (IP3) pathway. Centrally, ghrelin promotes feeding by directly enhancing the electrical activity of AgRP/NPY neurons. In the periphery, when ghrelin binds to GHSR, it suppresses the electrical activity of the gastric vagal afferent neurons transmitted to the NTS and hypothalamic AgRP/NPY neurons [18], inducing an orexigenic response. Based on this, and considering that NAR can cross the blood–brain barrier, its agonistic or antagonistic effects should be elucidated. Antagonists inhibit the excitatory effect, evident by suppressed c-Fos expression or immunoreactivity, a marker of neuronal activation in hypothalamic cells [78].

In the periphery, the GHSR has been shown to be involved in regulating gastrointestinal motility, according to evidence showing that peripheral administration of ghrelin-induced gastric emptying in animals. Although an in vitro study revealed that NAR induces calcium mobilization through GHSR, suggesting receptor activation [30], its in vivo effects remain to be fully determined. GHSR agonists have been found to enhance gastric emptying and alleviate symptoms associated with gastroparesis [79]; whether NAR exerts an antagonistic effect capable of delaying gastric emptying and further enhancing satiety is still unknown. Furthermore, non-peptide molecules are currently being investigated with orexigenic effects in vivo, which confirms the potential of other classes of molecules to regulate this receptor [80].

Ghrelin is the main appetite-stimulating (orexigenic) peptide, which is synthesized and secreted mainly by gastric PD/D1 or X/A (rats) cells. In healthy individuals, circulating levels of ghrelin increase during fasting and before the onset of feeding, followed by a postprandial decrease, thereby regulating short-term food intake, while low ghrelin levels have been documented in obese individuals [81]. Ghrelin also regulates the long-term induction of adiposity [11], apparently by activating the sympathetic nervous system, which increases fat deposition in adipose tissue and the liver [12]. Ghrelin itself can be regulated by macronutrients through an inhibitory effect on its circulating levels [82]. The absence of changes in serum ghrelin levels documented in the present study could be attributed to a lack of food intake, as samples were collected during a fasting period. Furthermore, the lack of significant differences between groups could be due to the duration of the experimental model, which may not have been sufficient to detect the ghrelin changes observed in other studies. For instance, in diet-induced obesity murine models lasting 9 to 12 weeks, total plasma ghrelin levels were found to be lower, as compared to controls [83,84,85]. These findings are associated with a decrease in gastric ghrelin mRNA levels and hypothalamic GHSR mRNA after exposure to an obesogenic diet [83,86]. In particular, the diet used by Holá et al. [84] contained a higher fat content (60%) and was based on a different animal species (mice) than the present study, which could explain the discrepancies in serum ghrelin levels. In contrast, the study of Yasrebi et al. [87] used a 12-week diet-induced obesity mouse model, and showed no significant differences in plasma ghrelin, as compared to the low-fat diet group, although differences in GHSR gene expression were observed at the central level [87]. These findings align with those of Briggs et al. [88], who demonstrated that central resistance to ghrelin develops in AgRP/NPY neurons 3 weeks after switching to a high-fat diet, as evidenced by the lack of immunoreactivity in them and the inability to generate a ghrelin-induced action potential [88]. These observations suggest that central changes, including GHSR desensitization secondary to decreased gene expression, precede changes in plasma ghrelin levels. This central desensitization has been proposed as a protective mechanism by the hypothalamus to prevent excessive positive energy balance [83]. Taken together, these studies indicate that a diet-induced obesity model of longer duration than 8 weeks may be necessary to effectively study ghrelin dysregulation at the peripheral level.

Lindqvist et al. [52] investigated the effects of fructose and found results consistent with a satiating effect, but differed regarding ghrelin levels. For this, rats were given a 23% fructose solution for 2 weeks and were shown to consume less food than animals given plain water. This reduced food intake was associated with a 40% increase in serum ghrelin levels in the fructose group, while no changes in ghrelin were observed in rats given sucrose 23%, glucose 23%, or plain water [52]. The increase in serum ghrelin in the Lindqvist [52] study is not consistent with the expected food consumption behavior, as ghrelin typically exerts an orexigenic effect at the central level. This discrepancy may be leptin-dependent, as ghrelin positively correlates with increased serum leptin [86]. Such a mechanism could explain the anorexigenic effect observed [88], although leptin was not analyzed in the present study. These results confirm that the physiological role of ghrelin is not limited to the execution of an orexigenic effect, and that other parallel effects must be considered, such as changes in lipid and carbohydrate metabolism, energy balance, and, in the long term, adiposity, as well as its interplay with leptin [19].

CCK can be detected in low basal concentrations that increase up to 5-fold after nutrient stimulation [89], such as lipids (mainly) and carbohydrates in the gut. Fatty acid chain length greater than C10 stimulates CCK secretion [90]. CCK decreases gastric emptying, thereby contributing to early satiety and decreased food intake or energy expenditure, and is therefore considered a short-term anorexigenic regulatory mechanism. This is the result of CCK binding to the peripheral CCKR in afferent vagal nerves, and the positive modulation of POMC/CART neurons, which promote central satiety and oppose the effect of AgRP/NPY neurons.

In lean individuals, postprandial CCK levels rise rapidly, reaching peak plasma concentration within 15 min of feeding, while obese individuals may experience either prolonged elevation or reduced postprandial CCK levels [9,91], leading to inconclusive findings regarding CCK levels in this population. A clinical study by Samra et al. [92] analyzed peptide hormones like GLP-1, CCK, and PYY in 37 obese (*n* = 16) and normal-weight adults (n = 21). After a 12 h fast, no significant differences in CCK levels were found between the groups. In contrast, a study by Ali Ahmad et al. [9] reports that 30 obese and 30 lean individuals underwent a 12 h fast, after which the lean individuals had significantly higher CCK levels than the obese group. These discrepancies between studies may be due to sample size. Therefore, CCK levels and their dysregulation in obesity require further study to establish a clear difference [89]. Nevertheless, it has been shown that there is a resistance to CCK that develops in obesity. This resistance allows a loss of its anorexigenic effects by reducing its receptor’s responsiveness [7,10], which manifests itself in hyperphagia that further promotes the obese state.

CCK-A receptors are located on vagal afferents, myenteric plexus, gallbladder, hypothalamus, and NTS. Centrally, CCKR inhibits the expression of orexigenic peptides and antagonizes the effects of ghrelin [8]. Peripherally, following its secretion by cells in the small intestine, CCK activates primary vagal afferent neurons in the GIT. Sulfated CCK, upon binding to CCK-A, activates signal pathways including protein kinase A, phospholipase C, phospholipase A2, and phosphatidylinositol 3-kinase, which evokes depolarizing currents, subsequent gating of transient receptor potential (TRP) channels and/or L-type voltage-activated calcium channels and induces calcium currents movements from extracellular origin. This results in the inhibition of vagally mediated gastric emptying and delayed gastric emptying, which further promotes satiety.

CCK1R null mice exhibit impaired satiation, thereby consuming more food. Peripheral CCK1R highlights a role in appetite control, making it a potential target for drugs that do not require crossing the blood–brain barrier to reach CNS-located receptors [93]. A challenge in developing effective CCK1R agonists for obesity treatment has been side effects since some have failed clinical trials, in addition to having minor or no additional benefits over acute dieting [93]. However, this research has led to the development of CCK receptor dual agonists [94].

A high-fat diet (HFD) has been shown to blunt CCK sensitivity and neuron activation [95]. Differences in CCK1R signaling and desensitization may exist depending on the location and expression in vagal afferent neurons, as well as the coupled signal transduction pathways [8]. At the cellular level, CCK1R signal desensitization could result from a loss of receptor function and signaling cascade and/or decreased function of the ion channel effector. Signaling studies have found that PKC could target the receptor desensitization process, whereas PKA signaling likely acts downstream of the receptor [8], although it is still unknown whether NAR is capable of regulating the CCK receptor in an agonistic manner.

Fat derived from a WD could initiate disruptions in CCK signaling. Consumption of HFD results in the loss of the feeding-suppressing effects of CCK, since dietary fat is one of the main stimuli for CCK secretion; continuous high levels of CCK lead to desensitization or downregulation of CCK-1R in the nodose ganglia and vagal afferent neurons [96,97]. Possible mechanisms associated with this desensitization include receptor internalization, sequestration, or post-receptor transduction cascades [97]. Reduced vagal sensing of nutrients and satiety hormones coincides with the onset of hyperphagia and increased body weight [96]. The time needed to develop reduced sensitivity to CCK in DIO rats appears to occur within the first 2 weeks. The mechanisms by which fat participates in this desensitization and fails to induce satiety are not entirely clear [97].

On the other hand, a high-fat/high-sucrose diet can significantly decrease gut innervation and induce vagal desensitization in the NTS, as evidenced by IB4 immunofluorescence staining [98], and may be linked to neurodegeneration secondary to microbiota dysbiosis, LPS production, gut and chronic inflammation. These changes are associated with an increase in body fat mass, independent of hyperphagia [98]. Further studies are essential to understand the mechanisms by which chronic inflammation and altered gut–brain vagal pathways may lead to obesity [98].

NAR demonstrated a dose-dependent increase in CCK secretion in STC-1 cells, which was associated with a mechanism of increased intracellular calcium concentrations through TRP ion channels [29]; in contrast, the glycoside form of NAR, naringin, did not elicit this same response [29]. This highlights the distinct ability of the aglycone form to promote enterohormone secretion, suggesting that it may have greater biological activity [24] secondary to differences in its chemical structure [99]. Although several studies have investigated the potential of different phenolics to stimulate CCK secretion in vitro and ex vivo [24,100,101], only a limited number have evaluated the capacity of naturally derived compounds to induce CCK secretion in vivo [102,103,104]. Furthermore, to date, no studies have investigated the in vivo effects of NAR on CCK secretion. In this regard, this study is the first, to the best of our knowledge, to identify a significant upregulation of CCK plasma levels in an in vivo model that was fed a standard diet in association with NAR supplementation, an effect that is abolished in a WD. These findings suggest that NAR correlates with CCK plasma level changes, which may be due to an upregulation of CCK synthesis by type I enteroendocrine cells; however, further studies are required to confirm this. Additionally, components of the diet are able to modulate CCK. In this regard, long-chain saturated fatty acids were found to be less stimulatory to CCK secretion [105]. This is important to note because the WD contained a significant amount of fat, mainly derived from lard, which is characterized by high saturated fat content [106]. Similarly, in the study conducted by Colley and Castonguay [58], a 16% fructose drink was given to rats over a 24 h period, which significantly decreased hypothalamic CCK expression by 37%, as compared to the control, while other beverages caused an increase (16% glucose, 16% high fructose corn syrup). Associated with this, a decrease in food consumption was observed in the fructose-exposed group, which is consistent with our findings. Although our model had a longer time frame than this study, dietary fructose may influence satiety through central CCK signaling. Taking this into consideration, the rats of the present study underwent a fasting period prior to blood sampling, which may have yielded different results on serum CCK levels, since CCK is a short-term regulatory hormone. Further studies that investigate CCK secretion at different time frames after feeding are warranted.

Previous studies have investigated the metabolism and benefits of orange juice consumption; however, there is a limited number of human studies evaluating the pharmacokinetics of NAR, as well as clinical trials that examine the therapeutic applications of pure, isolated NAR (as opposed to NAR in extract form).

Over the past decade, NAR administered at doses of 400 and 800 mg/kg body weight (b.w.) has demonstrated hepatoprotective effects and antioxidant properties in paracetamol-induced liver injury in vivo, providing evidence of NAR’s safety and efficacy in experimental models [107]. Doses ranging from 25 mg/kg [108] to 800 mg/kg b.w. [109] have been used in these models, with no adverse effects reported. Furthermore, in murine models, NAR has shown lethal dose 50 (LD50) values of over 2000 mg/kg b.w. with no adverse effects observed at doses below this threshold. This has led to classifying NAR as a “low toxicity” agent [110,111].

In clinical trials, oral doses ranging from 135 mg to 900 mg have been used [112,113,114,115], with no adverse effects reported, supporting NAR’s low toxicity and wide safety margin. For example, Kanaze et al. [112] used isolated NAR in a solid dispersion capsule (135 mg) in a pharmacokinetic study in 6 humans after a single oral administration, and none reported any adverse effects. In addition, Rebello et al. [114] verified the efficacy and safety of NAR in humans through a randomized, single-ascending dose study using capsules containing 150 mg of NAR derived from whole orange extract to evaluate doses ranging from 151 mg to 900 mg. No relevant adverse events or changes in blood safety markers were reported, following NAR ingestion. Based on another study conducted in primary human adipocytes, they suggest a dose of 300 mg every 12 h to achieve a physiological response.

Most pharmacokinetic studies of NAR have been conducted using animal models, which may initially serve as a predictive tool for bioavailability in humans. However, there could be significant differences in metabolic processes between species that may limit the accuracy of this correlation [116,117]. The aforementioned information highlights the need for additional pharmacokinetic studies in humans.

To the best of our knowledge, there is a limited number of studies that have evaluated the therapeutic effect of NAR. For example, a double-blind, placebo-controlled, randomized trial in 44 overweight and obese patients with NAFLD using a dose of 100 mg twice daily for 4 weeks (n = 22) [113,115] reported a decrease in BMI, visceral fat level, systolic blood pressure, and dyslipidemia; no adverse effects were reported. NAR might interact with drugs, as some in vivo studies have shown NAR to inhibit CYP3A4, CYP29, and CYP2E1 enzymes [118], resulting in inhibited metabolism of drugs like metoprolol, felodipine, and tofacitinib, leading to their accumulation [119,120,121]. However, further research is required to confirm this.

Most studies in animal models that have demonstrated the beneficial effects of NAR on various components of obesity have lasted up to 12 weeks [27], without reporting any adverse effects. Overall, the effects of NAR on metabolic parameters in both preventive and intervention obesity models suggest that it may be a promising protective agent, which warrants further investigation into the use of NAR, not only in obesity, but also in related disorders, such as metabolic syndrome, insulin resistance and/or NAFLD/MAFLD/MASLD. However, clinical trials with longer durations are required to better evaluate its therapeutic potential, as either a supplement or pharmacological agent, in order to further provide recommendations.

### Limitations

The present study has some limitations as follows:

(1) Time frame: Although 8 weeks is sufficient to induce obesity and observe early changes in metabolic parameters, some variables may require a longer time to provide deeper insight into changes, such as systemic inflammatory and OS changes, as well as changes in CCK and ghrelin. With this in mind, it is important to consider both acute and more advanced or chronic stages of the disease and document short- and long-term evidence;
(2) Another limitation is the lack of precise analysis of fructose intake, which could hinder the interpretation of results regarding energy intake and energy efficiency on adiposity;
(3) Collecting blood samples at multiple time points, including the fasting, prandial, and postprandial periods, could show dynamic changes in CCK and ghrelin levels in response to NAR treatment, offering a clearer understanding of their modulation through time;
(4) An analysis of inflammatory and OS markers in different adipose tissue deposits (e.g., visceral, epididymal, inguinal, subcutaneous) as well as the liver, could provide valuable insights into the effect of NAR on these tissues and their behavior during the experimental period, considering that inflammation affects each tissue during different time periods. Adipose tissue inflammation has been shown to occur prior to the onset of liver inflammation, which highlights the significance of exploring interventions that target inflammation in the early stages of this endocrine organ [122]. In addition, such analysis could help clarify the differences between adipose tissue depots, considering that abdominal obesity and visceral fat are risk factors for cardiovascular diseases [46];
(5) The need to confirm the predictive interactions and effect of NAR on the CCK receptors should also be considered, which could have an agonistic or antagonist effect, potentially promoting an orexigenic or anorexigenic effect. This could be achieved through the use of antagonists or in vitro models.

## 4. Materials and Methods

### 4.1. Ethical Approval and Animals

All experiments involving animals were reviewed and approved by the University of Sonora (UNISON) Research Ethics Committee (CEI-UNISON, No. 20/2023). Animal care was carried out in accordance with national regulations described in the Mexican NOM-026-ZOO-1999, as well as in the NIH care guidelines regarding the use and care of laboratory animals. Male Wistar rats (8 weeks of age, 278 ± 4 g) were obtained from the Department of Food Research and Graduate Studies of the UNISON (Hermosillo, Sonora, Mexico).

### 4.2. Experimental Design

For the WD-induced obesity model, 24 male Wistar rats underwent one week of acclimatization in individual metabolic cages under controlled conditions (25 °C; 40–70% humidity; 12 h light/dark cycles) and free access to a basal diet and water. The animals were then randomly assigned to one of four experimental groups (n = 6/group), that were fed the following non-isocaloric diets: Group 1 (BD) was fed a basal diet based on AIN 93G (15% energy from fat, 64% from carbohydrates, and 20% from protein); Group 2 (WD) was fed a Western diet with 36% energy from lipids (DYETS^®^ lard, Bethlehem, PA, USA 160 g, soybean oil 30 g; total 190 g), 47% energy from carbohydrates (starch 230 g, maltodextrin 70 g, sucrose 239.5 g; total 539.5 g), and 16% energy from proteins (casein 190 g). In addition, 20% fructose was given in the animals’ drinking water. The calculation of kcal/g of each experimental diet does not include the fructose included in the water. Kcal were determined based on the following equivalences: carbohydrates: 4 kcal per 1 g; lipids: 9 kcal per 1 g; protein: 4 kcal per 1 g; fiber (cellulose): 2 kcal per 1 g [123]. This diet was developed based on formulations reported by Reichardt et al. [124] and Bortolin et al. [125] (Table 4). Group 3 (WD + NAR) was fed the WD supplemented with NAR (Sigma-Aldrich, St. Louis, MO, USA); Group 4 (BD + NAR) was fed with a basal diet supplemented with NAR. NAR was administered at a dose of 100 mg/kg body weight, dissolved in 0.5% carboxymethylcellulose (CMC) as a vehicle, by oral gavage once daily at the same time for 8 weeks. The remaining groups were administered an equivalent volume of 0.5% CMC by oral gavage. The NAR treatment dose was determined based on previous studies and took into consideration subtoxic doses [40,111].

At the end of the experimental period, the animals were fasted for 6 h and anesthetized with pentobarbital (Aranda^®^ Aranda, Querétaro, Mexico; 60 mg/kg intraperitoneal). Blood samples were then collected by cardiac puncture in tubes with and without EDTA, from which plasma and serum were separated and stored at -80 for subsequent analysis. The liver and adipose tissue (abdominal and retroperitoneal) were extracted, and their weights were recorded.

The focus of this study was to elucidate the effect of NAR in a preventive model during the early stages of obesity. Although there is no clear consensus on the definition of “obesity” in animal models, any significant increase in the levels of a marker compared to the control group can be considered as obesity [37]. Additionally, the intervention time required for the development of obesity varies widely across studies and although a longer intervention is considered to consolidate the phenotypic and metabolic changes associated with obesity; it is estimated that phenotypic and physiological changes require 4 to 8 weeks in animal models [126] with a significant increase in body weight starting in the seventh week [44]. An 8-week obesity model was chosen since this period is considered the minimum necessary to establish obesity. Obesity was induced with a WD, which was supplemented with 20% fructose in the drinking water. This combination has been shown to have an aggravating and additive effect on the development of obesity, accelerating its onset within a shorter period of time [127,128].

### 4.3. Histological Analysis

The liver was washed with saline solution, divided into segments, immersed in 10% formaldehyde, and embedded in paraffin for further analysis. Samples were stained with hematoxylin and eosin (H&E) in order to determine hepatic steatosis.

### 4.4. Biochemical Analysis

Serum triglycerides (TG), total cholesterol, low-density lipoprotein (LDL-c), and high-density lipoprotein (HDL-c) were quantified using commercial kits (RANDOX) according to the manufacturer’s specifications. Very low-density lipoproteins (VLDL-c) were calculated based on TG (TG/5) [32,33].

### 4.5. Antioxidant Capacity Determination

Enzyme activities of superoxide dismutase (SOD) (CS0009, Sigma-Aldrich, St. Luois, MO, USA) and catalase (CAT) (EIACATC, Thermo Fisher, Waltham, MA, USA) were determined in plasma by colorimetric activity kits, according to manufacturers’ instructions.

### 4.6. Satiety Evaluation, In Vivo and In Silico

In order to evaluate the impact of diet on body weight and satiety, food intake was recorded daily, while body weight gain was measured every 4 days. Feed efficiency was also determined by using the formula: body weight gain (g/day)/energy intake (Kcal/day) [33,129], which evaluates the relationship between feed or energy intake and adiposity, or the percentage of consumed feed stored as fat [32,33]. This makes it possible to determine an association between these two variables.

Serum concentrations of cholecystokinin (CCK) and ghrelin were quantified using commercial enzyme immunoassay (ELISA) kits, based on the indirect competitive enzyme immunoassay principle, according to the manufacturer’s instructions (RayBiotech, Peachtree Corners, GA, USA). Absorbances were read on a spectrophotometer at 450 nm, and data were analyzed by a four-parameter logistic model (4-PL). Concentrations are expressed as pg/mL for CCK and ng/mL for ghrelin.

An in silico evaluation was conducted to determine the interaction between NAR, CCK, and ghrelin receptors. Ligand and receptor preparation was first performed. For this, the 3D structure of NAR was downloaded from the PubChem database (https://pubchem.ncbi.nlm.nih.gov/) (accessed on 19 February 2024), CID:932, in SDF format. All possible conformations were analyzed in M.O.E. (Molecular Operating Environment 2008.10) software. To explore the ligand binding sites in the 3D structures of CCK1R and GHSR, the crystal structures of the extracellular domain were obtained from the Protein Data Bank (PDB) database (http://rcsb.org) (accessed on 19 February 2024) (in PDB format) with identifiers (PDB ID): 7MBX for CCKR, and 7NA7 for GHSR, both of them selected based on resolution quality (<3 Å). Solvent (water) molecules were removed to avoid solvent-mediated interactions of the ligands with active and allosteric sites of the receptors. Also, unnecessary ligands were eliminated from the PDB structure: for CCKR (7MBX), the E chain (cholecystokinin) as well as A, B, C, and D chains (other ligands); for GHSR (7NA7), the A, B, C chains (receptor subunits) D, and F chains (other ligands). The molecules were subsequently protonated by adding hydrogens, and the energy was minimized using the AMBER99 (Assisted Model Building and Energy Refinement) parameters to facilitate the interaction and stabilize the structures.

### 4.7. Molecular Docking

After ligand and receptor preparation, molecular docking was performed using M.O.E. 2022.02 software [130]. Using the London dG scoring function [131], the possible interaction entries between the number of possible conformations of the molecules (“ligand and receptor preparation”) with the receptors of interest in the rigid state were retrieved. The results were sorted according to S-score in descending order. The interaction with the most negative value according to the S-score was selected, considering it as the prediction with the best interaction, best binding energy, and highest affinity [34,132]. The 2D interaction in the ligand–receptor binding site was subsequently analyzed by identifying the atoms and amino acids involved, as well as types of bonds, including hydrogen bonds, hydrophobic interactions, Van der Walls forces, and electrostatic interactions. Visualization of the interactions in 3D mode was performed in UCSF-CHIMERA (1.15) (https://www.cgl.ucsf.edu/chimera/) (accessed on 21 March 2024) [133].

### 4.8. Statistical Analysis

Results are expressed as mean ± standard error of the mean (SEM). Samples were analyzed in duplicate. To evaluate differences between treatments, an analysis of variance (ANOVA) was performed followed by Duncan’s test. A significant difference was considered when *p* < 0.05. Correlations between variables were calculated using Pearson’s correlation. Statistical analysis was performed using JMP 12.0 software and Infostat 2020.

## 5. Conclusions

Naringenin (NAR, 100 mg/kg, daily oral gavage) was administered as an antiobesity agent in a rat model of Western diet (WD)-induced obesity. NAR exerted an anti-obesogenic effect, according to reduced retroperitoneal adiposity, liver hepatosteatosis, and improvements to the lipid profile. NAR supplementation increased CCK levels when administered alongside a basal diet (BD); this effect was abolished in WD-fed animals. Total energy consumption did not change in any group, but feed efficiency decreased in the WD+NAR group. In silico, NAR appears to interact with CCK and ghrelin receptors (both of them involved in the hunger/satiety pathway), despite being a non-peptide molecule, potentially explaining part of its anti-obesogenic action. These interactions occur through binding with different amino acid residues in each receptor, which could facilitate their modulation. However, whether NAR acts as an agonist or antagonist remains to be fully determined. These findings contribute valuable insights into the action of NAR, which could support its therapeutic and/or preventive role as an anti-obesogenic.

## Figures and Tables

**Table 1:** Effect of NAR on energy intake and feed efficiency.

Variable	BD	WD	BD + NAR	WD + NAR
Energy intake (Kcal/day)	80.57 ± 1.56 ^a^	70.49 ± 2.79 ^b^	81.37 ± 1.85 ^a^	70.23 ± 3.12 ^b^
Feed efficiency (g/Kcal)	0.03 ± 0.002 ^c^	0.06 ± 0.002 ^a^	0.03 ± 0.002 ^c^	0.05 ± 0.002 ^b^

Feed efficiency (g/Kcal) was calculated as follows: body weight gain (g/day)/energy intake (Kcal/day). BD: basal diet; WD: Western diet; BD + NAR: basal diet + naringenin; WD + NAR: Western diet + naringenin. Data are presented as mean ± standard error of the mean (SEM) (n = 6 per group). Different letters represent significant differences between groups (*p* < 0.05). NAR: 100 mg/kg body weight.

**Table 2:** Characteristics of NAR interactions with the CCK (CCKR) and ghrelin receptors (GHSR). Negative bonding values based on the S-score, amino acid residues involved, behavior of NAR as electron donor or acceptor, and particular bonds obtained from the molecular docking.

Receptor	S-Score	Amino Acids	Donor	Acceptor	Particular Bonds
CCKR	−5.5540	Arg197, Asn194	1	0	Arg197: arene–cation with A ring; Asn194: OH of 7′ of phenolic A ring, as donor.
GHSR	−5.7476	Gln120, Asp99	1	1	Gln120: as sidechain donor to carbonyl in position 4 of the C ring; Asp99: as sidechain acceptor of hydroxyl group of 4′ of the B ring.

**Table 3:** Summary of the findings reported in the present work.

Variable	Effect of NAR Administration in the Setting of DIO with a WD
Body weight gain (g)	-
Retroperitoneal adipose tissue (g)	↓
Liver weight (g)	↓
Triglycerides (mg/dL)	↓
Total cholesterol (mg/dL)	↓
HDL-c (mg/dL)	↑
LDL-c (mg/dL)	↓
VLDL-c (mg/dL)	↓
Feed efficiency (g/Kcal)	↓
CCK (pg/mL)	-
Ghrelin (ng/mL)	-

“-” no significant effect; “↓” significantly decreased; “↑” significantly increased; HDL-c: high-density lipoprotein cholesterol; LDL-c: low-density lipoprotein cholesterol; VLDL-c: very low-density lipoprotein cholesterol; CCK: cholecystokinin.

**Table 4:** Composition of the experimental diets (%).

Ingredient/Component	BD	WD
Casein	20	19
Lard	0	16
Soybean oil	7	3
Starch	44.75	23
Maltodextrin	13.2	7
Sucrose	5	23.95
Fiber	5	3
Methionine	0.3	0.3
Choline	0.25	0.25
Vitamins	1	1
Minerals	3.5	3.5
Fructose	-	20% (dissolved in the drinking water)
Energy (Kcal/g)	4.06	4.70 *

BD: basal diet; WD: Western diet. * The calculation of kcal/g does not include the fructose included in the water. Kcal were determined based on the following equivalences: carbohydrates: 4 kcal per 1 g; lipids: 9 kcal per 1 g; protein: 4 kcal per 1 g; fiber (cellulose): 2 kcal per 1 g.

## Data Availability

The original contributions presented in this study are included in the article. Further inquiries can be directed to the corresponding author.
